# Comparative* In Vitro* Binding Studies of TiCl_2_(dpme)_2_, Ti(ada)_2_(bzac)_2_, and TiCl_2_(bzac)(bpme) Titanium Complexes with Calf-Thymus DNA

**DOI:** 10.1155/2015/836928

**Published:** 2015-12-30

**Authors:** Pamita Awasthi, Nitesh Kumar, Raj Kaushal, Mohan Kumar, Shrikant Kukreti

**Affiliations:** ^1^Department of Chemistry, National Institute of Technology, Hamirpur, Himachal Pradesh 177005, India; ^2^Department of Chemistry North Campus, University of Delhi, Delhi 110007, India

## Abstract

The binding of TiCl_2_(dpme)_2_ (**1**), (dpme = 6,6′-dimethyl-2,2′-bipyridine), Ti(ada)_2_(bzac)_2_ (**2**), (ada = adamantylamine; bzac = benzoylacetone), and TiCl_2_(bzac)(bpme) (**3**), (bpme = 4,4′-dimethyl-2,2′-bipyrdine) with calf thymus (ct) DNA has been studied by UV-visible spectroscopy, thermal denaturation, and circular dichroism spectroscopy. In UV-visible study complexes** 1**,** 2**, and** 3** showed red, blue, and red shifts, respectively, upon the addition of ct-DNA along with a significant hyperchromism. The intrinsic binding constants (*K*_*b*_) calculated from UV-visible absorption studies were 2.3 × 10^3^ M^−1^, 3.3 × 10^3^ M^−1^ and, 7.1 × 10^3^ M^−1^ for complexes** 1**,** 2**, and** 3**, respectively. The change in melting temperature (Δ*T*_*m*_) was calculated to be 2-3°C for each complex. Circular dichroism (CD) study showed blue shift for complex** 2** and red shift for complexes** 1** and** 3** along with rise in molecular ellipticity upon the addition of complexes. Results suggest a binding mode of complex** 2** different than** 1** and** 3**.

## 1. Introduction

The interaction of metal complexes with DNA is the current subject of investigation for the development of new class of drugs in the field of medicinal chemistry. Presently, number of studies in the area of transition metal complexes as antibacterial [1, 2] and anticancer agents [3, 4] and so forth are reported in literature. The role of transition metals to be anticancer drug has been explored after the discovery of cisplatin in 1969. Cisplatin was found much more effective against many types of cancer, particular testis, and ovary cancer [5–8]. It acts by forming covalent bonds with nucleophilic N7-sites of purine bases in DNA, exposed in the major groove of the double helix of DNA [8]. But due to its side effects, many efforts have been made worldwide to synthesize new novel nonplatinum anticancer drugs [9–12] with the hope of discovering new class of drugs with better chemotherapeutic effects and reduced side effects. After cisplatin the first nonplatinum anticancer drugs were titanium based budotitane and titanocene dichloride [13]. Later on many derivatives of budotitane and titanocene dichloride have been synthesized and tested on several cancer cell lines [14], but the mechanism of action of titanium complexes remained unclear till date. Primary target of majority of anticancer drugs is proposed to be DNA. Mechanism of action of cisplatin upon interaction with DNA is well proposed as it acts via groove binding [8]. We have synthesized different series of titanium complexes and carried out* in vitro* cytotoxic studies on HeLa, C6 and CHO cell lines [15, 16]. In present research paper, we have extended the binding studies of TiCl_2_(dpme)_2_, (**1**), Ti(ada)_2_(bzac)_2_, (**2**) and TiCl_2_(bzac)(bpme), (**3**) complexes (better IC_50_ values against HeLa cell line) with calf-thymus DNA with an objective to check the effect of ligands attached to the metal and type of mechanism followed upon interaction. Results indicate the different mode of interaction of** 1**,** 2**, and** 3** complexes with ct-DNA.

## 2. Materials and Methods

### 2.1. Materials

#### 2.1.1. Cell Culture

The cancer cell lines used for cytotoxicity study were cervical cancer cells (HeLa cell line), Chinese hamster ovarian cells (CHO), and rat glioma (C6 cell line) which were obtained from Indian Institute of Integrative Medicine (IIIM), Jammu, India. These cells were cultured in Dulbecco's Modified Eagle's Medium (DMEM) (pH 7.2–7.4) supplemented with fetal calf serum (10% FCS), penicillin (100 units/mL), and streptomycin (100 *μ*g/mL) at 37°C under a 5% CO_2_ humidified atmosphere. Cells were treated with different concentrations of complexes dissolved in dimethyl sulphoxide (DMSO) while the untreated control cultures received only DMSO for the calculation of IC_50_ value.

#### 2.1.2. MTT Assay

The cytotoxic activity of the synthesized complexes against the HeLa, C6, and CHO cell lines was determined using the colorimetric 3-(4,5-dimethylthiazol-2-yl)-2,5-diphenyl tetrazolium bromide (MTT) assay. This assay was carried out in triplicate in 96 well plates [15, 16]. Briefly, the cell pellet was resuspended in complete growth medium to get 1.5 × 10^5^ cells/mL and 100 *μ*L of cell suspension per well were seeded in tissue culture plate. Cells were treated with different concentrations of complexes and incubated for 12 h in a CO_2_ incubator (37°C, 5% CO_2_ and 90% relative humidity). Thereafter, 20 *μ*L of freshly prepared MTT solution (5 mg/mL in PBS, sterile filtered) was added to each well. Culture plates were gently stirred at 150 rpm for 5 min, to thoroughly mix the MTT into the media, and then incubated for 4 h at 37°C, to allow metabolization of MTT. MTT formazan crystals (MTT metabolic product) were resuspended in 100 *μ*L of DMSO. After then, the plates were stirred for 20 min in order to dissolve formazan crystals and the OD was measured with an ELISA reader (Biotek Synergy HT) at 570 nm.

#### 2.1.3. DNA Binding Experiments

Calf thymus DNA was purchased from S.G. enterprises and stock solution of DNA was prepared by dissolving an appropriate amount of DNA in double distilled water. Sodium cacodylate (pH = 7.4, containing 1.0 × 10^−5^ mol L^−1^ NaCl and 0.1 × 10^−3^ mol L^−1^ EDTA) was used as the buffer solution for making ct-DNA samples. The concentration of cacodylate buffer was 20 mM. It was found that solution of ct-DNA gave a ratio of UV absorbance at 260 and 280 nm more than 1.8, indicating that DNA was sufficiently free from protein. The titanium compounds were dissolved in DMSO and further diluted in Tris HCl buffer for making dilutions.

#### 2.1.4. Apparatus and Measuring Techniques

UV-visible spectra were measured on UV-1650 Shimadzu spectrophotometer in the range of 220 to 320 nm. CD spectra were recorded on JASCO J-815 spectrophotometer from 220 to 320 nm range, with the average of three scans. The DNA melting studies were done on CARY 100 spectrophotometer (Varian America) equipped with temperature controller.

#### 2.1.5. UV-Visible Measurements

Absorption experiments were carried out by keeping the titanium complexes concentration constant (10 *μ*M) while varying the ct-DNA concentration from 100 to 180 *μ*M. Absorption values were recorded after each successive addition of ct-DNA solution. The absorption data were analyzed and intrinsic binding constant (*K*_*b*_) was calculated.

#### 2.1.6. Thermal Denaturation Experiments

DNA melting study was carried out in the range of 10 to 90°C with 5°C difference by keeping the titanium complexes concentration 10 *μ*M and DNA concentration 100 *μ*M.

#### 2.1.7. Circular Dichroism (CD) Studies

In CD spectrophotometer the optimal chamber was deoxygenated with dry nitrogen before use. The CD spectra of ct-DNA incubated with titanium complexes at molar ratios ([titanium complex]/[ct-DNA]) of 0.1, 0.2, 0.3, 0.4, 0.5 were measured in the wavelength range of 220–320 nm. The changes in the spectra were monitored against a blank, that is, sodium cacodylate buffer.

#### 2.1.8. Preparation of Titanium Complexes

**Figure 1:**
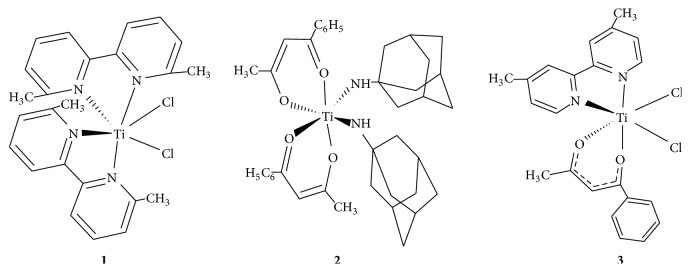
Proposed structure of titanium complexes.

The titanium complexes were synthesized by reacting titanium tetrachloride and nitrogen containing ligands in predetermined molar ratios. However, the detailed synthetic procedure and chemicals used for the synthesis of titanium complexes have been previously reported [15–17]. The proposed chemical structure of titanium complexes which were used for DNA binding studies has been shown in Figure 1.

## 3. Results and Discussion

### 3.1. Electronic Absorption Spectroscopy

**Figure 2:**
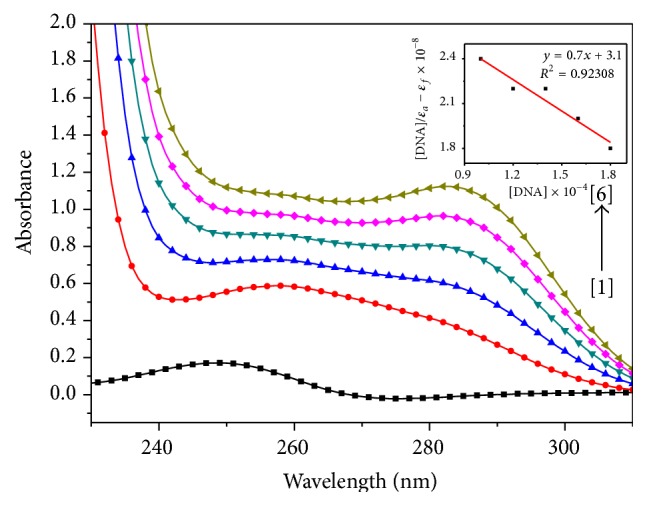
Absorption spectra of [TiCl_2_(dpme)_2_] upon addition of ct-DNA. [complex] = 10 *μ*M, [DNA] = (0) [1], (100) [2], (120) [3], (140) [4], (160) [5], (180) [6] *μ*M. Arrow indicates the absorbance changing upon the increase of DNA concentration. Inset: plot of [DNA]/(*ε*_*a*_ − *ε*_*f*_) versus [DNA] for the titration of ct-DNA with titanium complex.

**Figure 3:**
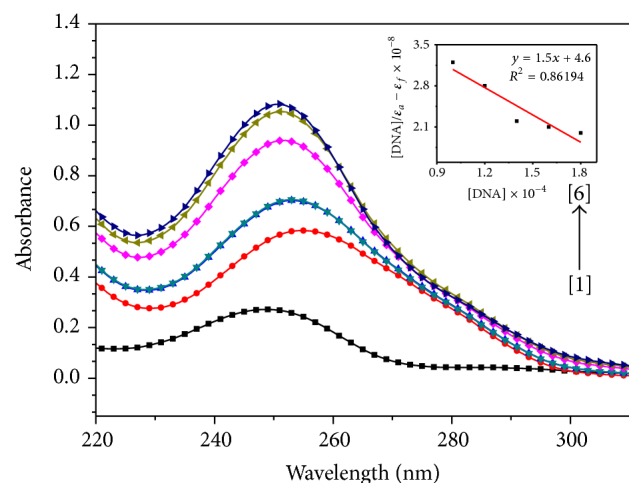
Absorption spectra of [Ti (ada)_2_(bzac)_2_] upon addition of ct-DNA. [complex] = 10 *μ*M, [DNA] = (0) [1], (100) [2], (120) [3], (140) [4], (160) [5], (180) [6] *μ*M. Arrow indicates the absorbance changing upon the increase of DNA concentration. Inset: plot of [DNA]/(*ε*_*a*_ − *ε*_*f*_) versus [DNA] for the titration of ct-DNA with titanium complex.

**Figure 4:**
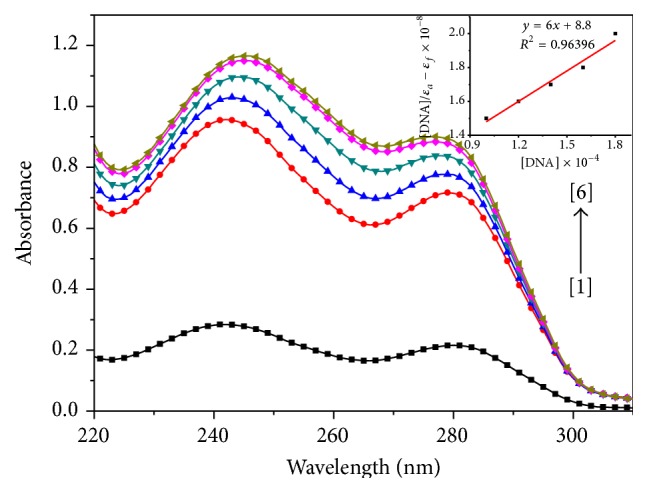
Absorption spectra of [TiCl_2_(bzac)(bpme)] upon addition of ct-DNA. [complex] = 10 *μ*M, [DNA] = (0) [1], (100) [2], (120) [3], (140) [4], (160) [5], (180) [6] *μ*M. Arrow indicates the absorbance changing upon the increase of DNA concentration. Inset: plot of [DNA]/(*ε*_*a*_ − *ε*_*f*_) versus [DNA] for the titration of ct-DNA with titanium complex.

The absorption spectra were recorded for a fixed concentration of titanium complexes** 1**,** 2**, and** 3** (10 *μ*M) with increase in concentration of ct-DNA (10–180 *μ*M) (Figures 2, 3, and 4). In the absorption spectrum of complex** 1**, we observed typical hyperchromic effect after complex binds to ct-DNA, with *λ*_max_ shift from 270 to 290 nm (Figure 2). Some changes in the structure of titanium complex as well as DNA have been observed during the titration. The structure of the titanium complex seems to get modified and modified structure is interacting with ct-DNA. As ct-DNA absorbs strongly at 260 nm, but with increasing ct-DNA in the solution of a titanium complex, there was a shift in wavelength from 260 to 280 nm as shown in Figure 2. The absorption spectrum of complex** 2** shows absorption band at 250 nm (Figure 3). With the addition of ct-DNA to titanium complex, the absorption gets red shifted along with hyperchromic effect. Further addition of ct-DNA causes hyperchromic effect along with a slight blue shift. This type of behaviour indicates the change in conformation of DNA upon interaction with metal complex [18]. It is reported in literature that hypochromism results from contraction of the DNA helix axes as well as conformational change in the molecule of DNA; however, hyperchromism results from secondary damage of DNA structure [19]. The observed hyperchromism effect can be due to electrostatic interaction between positively charged titanium compound and the negatively charged phosphate backbone at the periphery of double helix ct-DNA [20]. Initially complex** 2** gets stabilized following the same process as that for complex** 1** but due to the presence of bulky adamantylamine and benzoylacetone it gets destabilized. The absorption spectra of complex** 3** show absorption band at 240 and 280 nm (Figure 3). It has been observed that, with the increase in addition of ct-DNA to this titanium complex (complex** 3**), the absorption band at 240 nm gets shifted to 245 nm along with a significant hyperchromic effect. However, the absorption at 280 nm does not show any shift in wavelength. The extent of the hyperchromism is indicative of partial or nonintercalative binding modes, such as electrostatic forces, Vander Waals interaction, hydrogen bonds, and hydrophobic interaction. The results suggested that the mode of complex binding to ct-DNA involves a strong stacking interaction between base pairs of DNA and aromatic groups of complex. The intrinsic binding constant (*K*_*b*_) of all the three complexes was calculated through a plot of [ct-DNA]/*ε*_*a*_ − *ε*_*f*_ *versus* [ct-DNA] by using Wolfe-Shimmer equation (see (1)) from spectral titration data [21, 22]:

(1) $$\begin{array}{c} \frac{\left[DNA\right]}{\varepsilon_{a}-\varepsilon_{f}}=\frac{\left[DNA\right]}{\varepsilon_{b}-\varepsilon_{f}}+\frac{1}{K_{b}\left(\varepsilon_{b}-\varepsilon_{f}\right)}, \end{array}$$

where [DNA] is the concentration of ct-DNA, *ε*_*a*_ is the apparent extinction coefficient, *ε*_*f*_ corresponds to extinction of complex in its free form, and *ε*_*b*_ refers to extinction coefficient in bound form. For the calculation of binding constant (*K*_*b*_), extinction coefficients were determined at the wavelength of maximum absorbance in all the three complexes. In complexes** 1**,** 2**, and** 3** extinction coefficients were found out at 283, 252, and 245 nm, respectively. The value of intrinsic binding constant (*K*_*b*_) was calculated to be 2.3 × 10^3^ M^−1^, 3.3 × 10^3^ M^−1^ and 7.1 × 10^3^ M^−1^ for complexes** 1**,** 2**, and** 3**, respectively. The results support the fact that the complexes bind to DNA with different binding affinity [23]. The higher binding constant (*K*_*b*_) value obtained for complex** 3** suggests that more base pairs were made available for binding than those in complexes** 1** and** 2**. The higher binding affinity of complex** 3** is consistent with its higher cytotoxic activity against HeLa cell line [24]. Complex** 3** was found to be more effective against HeLa cell line with an IC_50_ value of 3.3 *μ*M, mentioned in our previous study [16]. The IC_50_ values of complexes [15, 16]** 1**,** 2**, and** 3** are tabulated in Table 1. Our results corroborate with Pd(II) 2,2′-bipyridine complexes binding to ct-DNA, hyperchromic effect is in same line, and binding constant (*K*_*b*_) value was also of almost same magnitude (*K*_*b*_ value of Pd(II) 2,2′-bipyridine complex was 3.78 × 10^3^ M^−1^) [25]. The observed hyperchromism in all the three complexes can be due to the presence of noncovalent interactions, that is, electrostatic interactions, hydrogen binding, and groove binding (major or minor) with outside the ct-DNA structure recommends the binding of complexes and stabilisation of ct-DNA with complexes [26].

### 3.2. Thermal Denaturation Study

**Figure 5:**
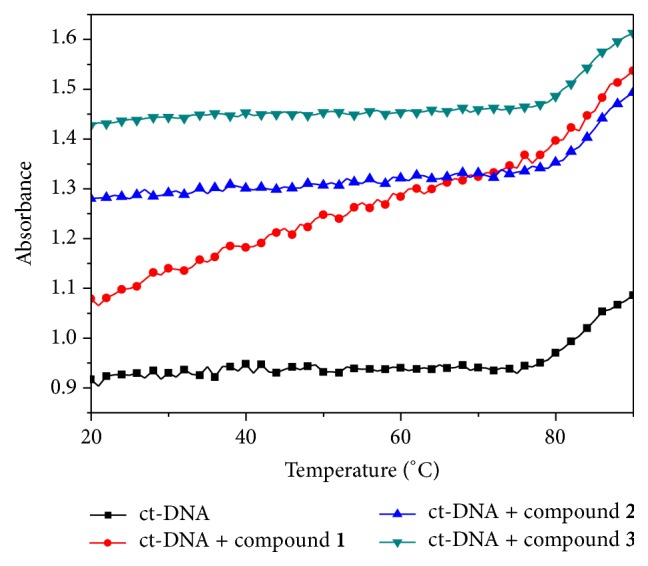
Melting curves of ct-DNA (100 *μ*M) in the absence and in the presence of titanium complexes (10 *μ*M).

The stability of complex** 1**/**2**/**3** + ct-DNA was further confirmed by DNA melting profile. We have observed the melting temperature of DNA alone and melting temperature of TiCl_2_(dpme)_2_/Ti(ada)_2_(bzac)_2_/TiCl_2_(bzac)(bpme) + ct-DNA complex (*D*/*N* = 0.1). The change in melting temperature (Δ*T*_*m*_) was found to be nearly 3°C as shown in Figure 5 (change in meting temperature was calculated by Δ*T*_*m*_ = *T*_*m*_ − *T* ^0^_*m*_, where, *T*_*m*_ and *T* ^0^_*m*_ are melting temperatures in the presence and absence of titanium complexes, resp.). The melting point was determined by observing change in absorbance. ct-DNA showed change in absorbance at 75°C, while TiCl_2_(dpme)_2_/Ti(ada)_2_(bzac)_2_/TiCl_2_(bzac)(bpme) + ct-DNA showed change in absorbance at around 78°C. It has been found that there was change in melting temperature of around 3°C. This small change in melting temperature of ct-DNA could be referred to groove interaction of titanium complexes at the backbone of ct-DNA [27], since it is well known that groove binding and electrostatic binding with phosphate backbone of DNA results in a little change in melting temperature, whereas intercalation mode of binding gives rise to a significant change in melting temperature of DNA double helix [28]. Hence the possibility of intercalation mode is ruled out.

### 3.3. Circular Dichroism (CD) Studies

**Figure 6:**
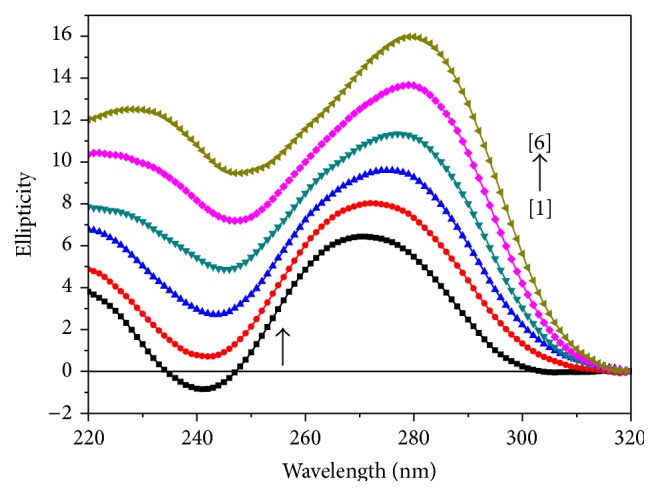
Circular dichroism spectra of ct-DNA (100 *μ*M), in the presence of increasing amounts of compound** 1** at the [DNA] = 100 *μ*M, [complex] = (0) [1], (10) [2], (20) [3], (30) [4], (40) [5], (50) [6] *μ*M.

**Figure 7:**
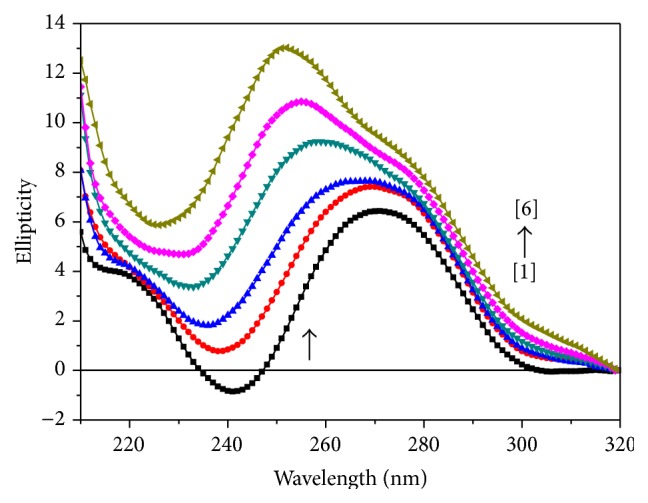
Circular dichroism spectra of ct-DNA (100 *μ*M), in the presence of increasing amounts of compound** 2** at the [DNA] = 100 *μ*M, [complex] = (0) [1], (10) [2], (20) [3], (30) [4], (40) [5], (50) [6] *μ*M.

**Figure 8:**
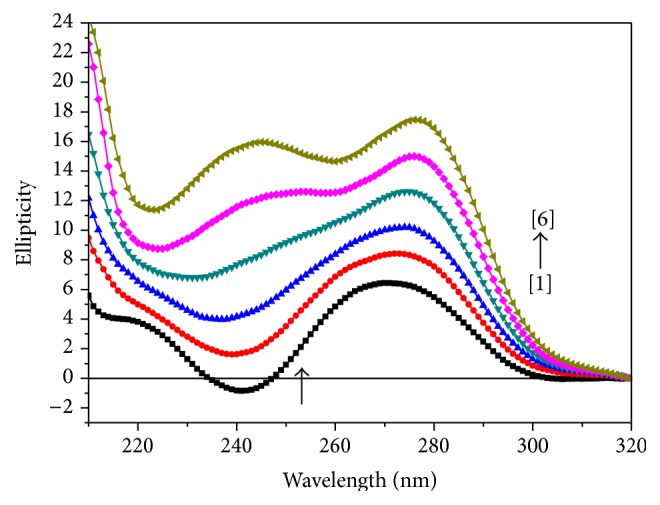
Circular dichroism spectra of ct-DNA (100 *μ*M), in the presence of increasing amounts of compound** 3** at the [DNA] = 100 *μ*M, [complex] = (0) [1], (10) [2], (20) [3], (30) [4], (40) [5], (50) [6] *μ*M.

The ct-DNA shows two conservative CD bands in the UV region: a positive band at 270 nm due to base stacking and a negative band at 240 nm due to right handed helicity of DNA. Changes in the CD spectra of DNA in the presence of complex** 1**,** 2**, and** 3** are shown in Figures 6, 7, and 8, respectively. The shapes of CD spectra are dependent on the concentration of added complexes (**1**,** 2**, and** 3**). When ct-DNA was incubated with metal complexes (**1**,** 2**, and** 3**), rise in molecular ellipticity was observed in both positive and negative ellipticity bands. The intensity of negative band (decreased) gets shifted towards zero level or above zero level and intensity of positive band (increased) showed significant hyperchromic shift in all the three cases (Figures 6, 7, and 8). Upon incubation of DNA with the metal complex** 1,** an increase in the molecular ellipticity values of both the positive and negative ellipticity bands along with a red shift of approximately 10 nm was shown. It has been observed from the spectrum (Figure 6) that as we have increased the concentration of complex** 2** in ct-DNA, a blue shift of approximately 8–10 nm has been observed. However, when ct-DNA was incubated with metal complex** 3** (Figure 7), rise in molecular ellipticity was observed in both positive and negative ellipticity bands along with red shift of approximately 6-7 nm. This may be due to the fact that DNA binding of titanium complexes affects the conformational changes of DNA. This conformational change is attributed to electrostatic interaction both along the phosphate backbone and between sites on the bases having partial negative charge and the negatively charged phosphate groups. Some investigators believed that this type of changes in the CD spectra may be reflected of a shift from a B-like DNA structure toward one with some contributions from an A-like conformation [29], but this phenomenon could be due to groove binding that stabilizes the right-handed B form of DNA [30]. This enhancement of the CD band of DNA at around 270 nm is due to distortions induced in the DNA structure [31].

## 4. Conclusions

In the present study we have reported the ct-DNA binding studies of previously reported titanium complexes. DNA binding studies were carried out using absorbance spectroscopy, circular dichroism, and thermal denaturation techniques. From these studies, it has been concluded that titanium complexes that seem to bind to DNA result in distortion in its structure as per UV-visible and melting behaviour of the complexes, whereas CD study indicates that compound** 2** behaves differently than** 1** and** 3**. Further, type and stereochemistry of the ligands do play an important role in target identification and interaction.

## Figures and Tables

**Table 1:** The IC_50_ values of titanium complexes on HeLa, C6, and CHO cancer cell lines as determined by MTT assay [15, 16].

Complex	Cell line (source)		
	Hela (cervical)	C6 (rat glioma)	CHO (ovary)
	IC_50_ (*µ*M)		
TiCl_2_(dpme)_2_ (1)	9.1	23	29.2
Ti(ada)_2_(bzac)_2_ (2)	4.06	21.8	46.1 [15]
TiCl_2_(bzac)(bpme) (3)	3.3	141.7	23.9 [16]
